# Three-Dimensionally Printed Vaginal Rings: Perceptions of Women and Gynecologists in a Cross-Sectional Survey

**DOI:** 10.3390/pharmaceutics15092302

**Published:** 2023-09-11

**Authors:** Laura Andrade Junqueira, Francisco José Raposo, Geraldo Sérgio Farinazzo Vitral, Atabak Ghanizadeh Tabriz, Dennis Douroumis, Nádia Rezende Barbosa Raposo, Marcos Antônio Fernandes Brandão

**Affiliations:** 1 Center for Research and Innovation in Health Sciences, Department of Pharmaceutical Science, Federal University of Juiz de Fora, Juiz de Fora 36036-900, MG, Brazil; laura.deandrade@hotmail.com (L.A.J.); ffox3000@gmail.com (F.J.R.); marcos.brandao@ufjf.br (M.A.F.B.); 2 Woman Health Investigation Group, Department of Surgery, Federal University of Juiz de Fora, Juiz de Fora 36036-900, MG, Brazil; geraldovitral@yahoo.com.br; 3 Centre for Innovation and Process Engineering Research, University of Greenwich, Chatham Maritime, Chatham ME4 4TB, UK; ata_ghanizadeh@hotmail.com (A.G.T.); d.douroumis@greenwich.ac.uk (D.D.)

**Keywords:** vaginal ring, 3D printing, personalized medical devices, fused deposition modeling, women’s health, drug delivery

## Abstract

Three-dimensional printing technologies can be implemented for the fabrication of personalized vaginal rings (VRs) as an alternative approach to traditional manufacturing. Although several studies have demonstrated the potential of additive manufacturing, there is a lack of knowledge concerning the opinions of patients and clinicians. This study aimed to investigate the perception of women and gynecologists regarding VRs with personalized shapes. The devices were printed with different designs (traditional, “Y”, “M”, and flat circle) by Fused Deposition Modeling for a cross-sectional survey with 155 participants. Their anticipated opinion was assessed through a questionnaire after a visual/tactile analysis of the VRs. The findings revealed that most women would feel comfortable using some of the 3D-printed VR designs and demonstrated good acceptability for the traditional and two innovative designs. However, women presented multiple preferences when the actual geometry was assessed, which directly related to their age, previous use of the vaginal route, and perception of comfort. In turn, gynecologists favored prescribing traditional and flat circle designs. Overall, although there was a difference in the perception between women and gynecologists, they had a positive opinion of the 3D-printed VRs. Finally, the personalized VRs could lead to an increase in therapeutic adherence, by meeting women’s preferences.

## 1. Introduction

The vaginal route has been used for centuries, initially for local effects and more recently to promote systemic responses [1]. It presents advantages such as having a highly vascularized surface area, avoiding the first-pass effect of drugs in the liver, bypassing gastrointestinal effects, and allowing for the administration of drugs with low oral bioavailability [2,3,4]. Currently, there are several dosage forms for vaginal delivery, including solution, emulsion, suspension, cream, gel, suppository, tablet, vaginal ring (VR), and film [5]. Semisolid products are traditionally used for a short period with repeated applications [6,7], while VRs are commonly used for drug delivery over prolonged periods (weeks to months) [6].

VRs are flexible polymeric devices that promote the controlled release of drugs over an extended period and can be used to induce local or systemic effects [8,9]. The ring is placed in the vagina by the woman herself with or without the aid of an applicator [10]. Currently, there are several marketed vaginal rings such as Estring^®^ and Femring^®^, used for hormone replacement therapy, and Progering^®^, Fertiring^®^, Annovera^®^, Nuvaring^®^, and Ornibel^®^, which are contraceptives [11]. Another close-to-market VR contains dapavirine for the prevention of HIV [12,13]. There are also a number of VRs under development for combined therapies such as the prevention of HIV, contraception, and/or prevention/treatment of other sexually transmitted diseases [14,15].

Although VRs present several advantages (safety, local application, few adverse effects, controlled drug release, and good patient compliance due to the low frequency of administration) [16], they are fabricated with a fixed geometry (circle) at the same sizes and doses without taking into account that women have different needs, habits, preferences, and physical characteristics [17]. Montgomery et al. (2019) [18], for example, evaluated the preference of women regarding four vaginal dosage forms and demonstrated that there was no clear favorite, showing the necessity of a range of options for end users. Therefore, the development of personalized VRs could lead to therapeutic benefits to promote women’s health. However, tailored vaginal devices can hardly be manufactured by conventional technologies (e.g., injection molding) [19].

Current applications are based on the “one-size-fits-all” approach, where patients receive the same medicines in similar doses and frequencies [20]. The existing manufacturing technologies are designed for large-scale production, making them cost-effective but allowing no space for dose variability [21]. Three-dimensional printing (or additive manufacturing) has been introduced as an innovative technology that could enable the transition from the current batch manufacturing approach to personalized medicines [22,23,24,25,26]. Objects in 3D printing are produced in a layer-by-layer manner based on a 3D digital design [27]. Due to its unlimited capabilities, 3D printing facilitates the fabrication of personalized doses with the customization of the drug release profiles, size, shape, and physical appearance through the production of small batches tailored to meet the needs of individual patients [28,29]. Moreover, digital control over the arrangement of matter provides a new level of freedom and flexibility for dosage form design. Due to the vast versatility of 3D printing, it has transformed into a highly desirable manufacturing method for the production of complex (external and internal) geometries, fabricating medicines with unique characteristics [30,31].

Three-dimensional printing is a promising technology to advance the manufacturing of personalized VRs (dosage, release profile, size, and shape) with complex geometries that promote functional improvements and meet women’s needs and preferences, increasing their acceptability and adherence to therapy. There are several reported studies on the use of 3D printing for the design and fabrication of VRs. Welsh et al. (2019) [32] employed the ARBURG plastic free-forming 3D printing technique to manufacture VRs loaded with dapivirine. Janusziewicz et al. (2020) [33] introduced a new approach to design and manufacture VRs with geometrically complex internal architectures using digital light synthesis. Arany et al. (2021) [34] used FDM to produce VRs manually filled with jellified metronidazole or chloramphenicol for the treatment of bacterial vaginosis. Koutsamanis et al. (2021) [35] investigated innovative polyester-based thermoplastic elastomers for the FDM 3D printing of VRs containing progesterone. Chen et al. (2022) [36] described the fabrication of reservoir-type VRs through FDM, enabling the controlled delivery of multiple active drugs. Tiboni et al. (2021) [16] fabricated clotrimazole VRs designed for the treatment of recurrent vaginal candidiasis. Fu et al. (2018) [17] investigated the 3D printing of vaginal rings with personalized shapes for the controlled release of progesterone.

Despite advances in 3D printing of VRs, the opinion of women (end users) and gynecologists (primary health care professionals responsible for prescriptions) regarding the concept of 3D-printed VRs with unique features has not yet been investigated. On the contrary, there are several published reports focusing on the viewpoints of various cohorts regarding 3D-printed oral dosage forms. For instance, Goyanes et al. (2017) [37] evaluated how the shape, size, and color of 3D-printed tablets influenced end-user acceptability. Bracken et al. (2022) [38] delved into the acceptability of 3D-printed tablets among children and young individuals. Fastø et al. (2019) [39] concentrated on patients with polypharmacy, examining the perceptions, preferences, and acceptability of this group toward 3D-printed medicines. Finally, Rautamo et al. (2020) [40] and Goh et al. (2022) [41] investigated the perceptions of healthcare professionals on 3D-printed tablets.

In therapy, patients play an essential role in achieving the desired effect, as they are in charge of controlling their medication. Thus, to facilitate drug administration and overcome the possible occurrence of problems (such as poor adherence and effectiveness of the treatment), a patient’s needs and preferences should be considered in the design of the pharmaceutical product [42]. As a consequence, such an approach can increase patients’ acceptability, which has a significant and positive impact on patient adherence and can improve their quality of life [43,44].

Therefore, the present work aims to investigate the perceptions of women and gynecologists regarding four different designs of placebo VRs produced by FDM 3D printing (no clinical evaluation). We initially evaluated whether women would feel comfortable using some of the geometries. Then, we assessed the women’s anticipated acceptability and preference and how the flexibility of the VR would impact their opinions. In addition, an investigation of how personal background could affect women’s acceptability was performed. For the gynecologists, we evaluated their willingness to prescribe each design, preference, and technical opinion about several parameters (suitability with the vaginal anatomy, level of difficulty for insertion and manipulation, comfort during use, and possible interference during sexual intercourse). Finally, the impact of the method of participation (in person or online) on the perceptions of women and gynecologists was studied.

## 2. Materials and Methods

### 2.1. Materials

Filaments (1.75 mm in diameter) of polyvinyl alcohol (PVA) and polylactic acid (PLA) were purchased from Shenzhen Esun Industrial Co., Ltd. (Shenzhen, China) and used to fabricate the VRs. According to the manufacturer, the PVA filament has a density of 1.25 g/cm^3^, a tensile strength of 22 MPa, and an elongation at break of 360% [45]. The PLA filament has a density of 1.23 g/cm^3^, a tensile strength of 63 MPa, and an elongation at break of 20% [46].

### 2.2. Design and 3D Printing of the VRs

**Figure 1:**
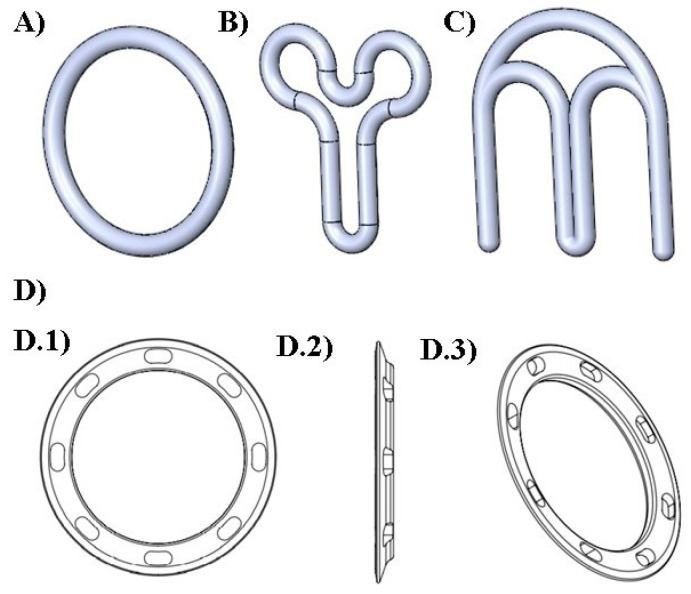
Digital designs of the different geometries of VRs produced. (**A**) traditional; (**B**) “Y”; (**C**) “M” (**D**) flat circle; views of the flat circle design: (**D.1**) front, (**D.2**) left side, and (**D.3**) perspective.

In this study, four different designs were selected to fabricate the VRs (Figure 1). The first design (named “traditional”) represented the commercial circular (or torus) shape. As shown in Figure 1, the second “Y” and the third “M” geometries previously proposed by Fu et al. (2018) [17] were also investigated. In addition, an innovative fourth design (named “flat circle”) was introduced for the functional improvement of VRs.

The VR designs were developed using computer-aided design (CAD) software (SolidWorks^®^ 2015 (Dassault Systèmes SolidWorks Corporation, Waltham, MA, USA)). FDM was selected as the 3D printing technology to fabricate the VRs, and a Dreamer NX (FlashForge, Jinhua City, Zhejiang Province, China) printer was used. Filaments of PVA and PLA were chosen due to their degrees of flexibility in producing flexible and rigid VRs, respectively. The printing parameters were set as follows: extrusion temperature, 205 °C; platform temperature, 50 °C; layer height, 0.18 mm for PVA and 0.21 mm for PLA; printing speed, 20 mm/s for PVA and 80 mm/s for PLA; infill, 30%; infill pattern, line.

### 2.3. Study Design

A cross-sectional, quantitative, single-site survey was approved by the Ethics Committee of the Federal University of Juiz de Fora (protocol number 4.743.407). The objective of the survey was to evaluate the perceptions of two groups (no clinical evaluation), women and gynecologists, regarding 3D-printed VRs. The questionnaires were completed in person or online. The final number of participants was 155.

### 2.4. Participants

The recruitment of participants (women and gynecologists) was carried out through an active search by the research team through propagation on social media and tracking in the University and University Hospital environment (Federal University of Juiz de Fora). The participants of the study were women aged 21–50 years and doctors (male and female) specializing in gynecology aged between 30 and 65 years.

### 2.5. Intervention

All participants signed a consent form. A member of the research team demonstrated various VR features (their use, how to insert them, and major advantages, mainly in relation to the frequency of administration), including the rationale of 3D printing technology. Subsequently, the 3D-printed devices were presented separately to the participants, who were able to visually observe the devices. The participants were asked to hold (flexible and rigid device) and fold (flexible device) the rings as they would for vaginal insertion. Eventually, the participants completed the printed hard copies of the questionnaire to express their opinions about the devices. For the online participation, the participants received a video via email or social media containing the same guidelines, and a copy of the questionnaire (Google Forms).

### 2.6. Questionnaire

The present work involved two different structured questionnaires; the first for the group of women and the second for the group of gynecologists. The sections of the questionnaires are described below.

#### 2.6.1. Sociodemographic Data

The initial section of both questionnaires included questions about sociodemographic data. The women were asked about their age, education level, income, and previous experience with the vaginal delivery of drugs. Gynecologists were asked about their age and sex.

#### 2.6.2. Comfort with Using 3D-Printed VRs

The women were questioned whether they would feel comfortable using one of the ring designs through 5-point Likert scale statements where they rated their level of satisfaction (ranging from “I strongly disagree” to “I strongly agree”).

#### 2.6.3. Acceptability and Preference

The European Medicines Agency defines acceptability as the overall ability and willingness of the patient to use the medicine as intended [47,48]. Preference describes the option of choice for product attributes (such as color, odor, viscosity, shape), dosage forms (tablets, implant, VR, gel), delivery routes, or even modes of administration that best suit the individual profile [49]. In the context of vaginal dosage forms, acceptability and preference are usually evaluated based on the actual use of a drug product (prior real-life experiences or clinical trials) [50,51,52] or a hypothetical product that can be shown as images or descriptions, or even handled during interviews and focus groups [53,54,55]. Since the participants in the present study did not use the VRs, we assessed anticipated acceptability and preference. For acceptability, the willingness to use (or to prescribe) was evaluated, where the participants were asked about the likelihood that they would use/prescribe each geometry using a five-point Likert scale (ranging from “I would not use/prescribe at all” to “I would definitely use/prescribe”). For preference, the participants were requested to order the geometries according to their preference, so that number 1 was the one they most liked and number 4 was the one they least liked.

#### 2.6.4. Flexibility

The overall VR flexibility was evaluated through 5-point Likert scale statements where the participants rated their level of agreement (ranging from “I strongly disagree” to “I strongly agree”) with the statement “The flexibility of the VR influences my willingness to use/prescribe it”. The participants then chose which type of flexibility they preferred (flexible or rigid).

#### 2.6.5. Gynecologists’ Technical Opinions

The technical opinion of gynecologists on each geometry was assessed regarding the suitability of the vaginal anatomy, the level of difficulty for insertion and manipulation, comfort during use, and possible interference during sexual intercourse. A nominal scale (yes, maybe, no) or a five-point Likert scale (ranging from “very difficult” to “very easy”) was used.

### 2.7. Statistics

Descriptive statistics are reported as the mean and standard deviation (quantitative variables) or median (1st quartile–3rd quartile) (categorical variables). The variables measured by the Likert scale were transformed into numeric values (1 = “I strongly disagree”, “I would not use/prescribe at all”, 5 = “I strongly agree”, “I would definitely use/prescribe”). Values 4 and 5 were considered criteria for acceptance of the geometry by users and for prescription by physicians. Differences between the in-person and online groups were assessed using the chi-square test (χ^2^) for nominal variables and the Mann–Whitney U test for ordinal variables. Student’s *t*-test for independent samples was used to test differences in the participants’ ages. The effect size (ES) was evaluated by Cramer’s V, adopting the following classification: small, <0.30; moderate, 0.30–0.49; large, ≥0.50 [56]. Analyses were performed using IBM SPSS software version 22.0 (IBM Corporation, New York, NY, USA). Differences were considered significant if *p* < 0.05.

## 3. Results and Discussion

### 3.1. 3D Printing of the VR Designs

**Figure 2:**
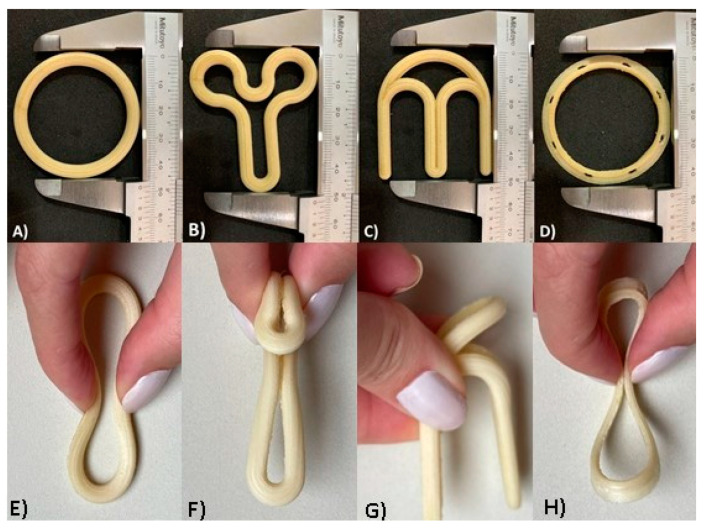
Optical images of the different VR designs produced by FDM technology with PVA filament: (**A**) design traditional; (**B**) design “Y”; (**C**) design “M”; (**D**) design flat circle. The VRs were folded as they would be for vaginal insertion: (**E**) traditional design folded; (**F**) “Y” design folded; (**G**) “M” design folded; (**H**) flat circle design folded.

As shown in Figure 2, the VR designs were fabricated by using FDM printing technology with filaments comprising PVA and PLA polymers. The devices fabricated using PLA (Supplementary Materials) were visually similar, with only a color variation. The rationale for the selection of PLA and PVA to print the VRs was to investigate the influence of the device’s flexibility on the acceptance criteria of women and gynecologists. The VRs fabricated with PLA were rigid, while PVA devices were flexible.

Despite the advantages of the existing VRs, they present some limitations, including increased vaginal secretions, potential for involuntary expulsion, differences in drug absorption, and cultural sensitivities [57,58]. With the objective of overcoming some of these drawbacks and taking into account the flexibility provided by 3D printing in the production of complex shapes, a new design (flat circle) was introduced for the first time in the present study. This innovative design (Figure 1D and Figure 2D,H) incorporates three fundamental structural enhancements compared to traditional VRs. Firstly, it features a notably more delicate cross-sectional profile, ensuring a gentle adaptation to the mucosal surface and promoting superior genital comfort. Secondly, we have introduced meticulously designed slopes within the cross-section to optimize fit, mitigating the risk of involuntary expulsions, and simultaneously augmenting the contact area with the mucosa to facilitate drug release. Lastly, strategically integrated “holes” within the cross-sectional structure serve to facilitate the passage of biological fluids, such as semen, menstrual flow, and vaginal secretions.

The physical characteristics of a medical device (sensorial properties and their mode of application) can affect women’s acceptance, especially for drug-eluting vaginal products. Shape, color, and texture are relevant characteristics to consider during the development of vaginal formulations. The shape has particular importance since it may impact the perception of the ease of application [49]. In the literature, the influence of the shape of vaginal suppositories on the willingness to try and the preference of women has been demonstrated [59,60]. Moreover, the design aspects of vaginal applicators have been reported to influence acceptance among target users [61]. In this study, the capability of 3D printing for the fabrication of VRs with complex shapes and different geometries was also taken into account. It was hypothesized that the geometry could potentially have a significant influence on women’s acceptability since the shape may affect the insertion and manipulation of the device as well as during sexual intercourse.

### 3.2. Evaluation of Women’s Perceptions

#### 3.2.1. Demographics of the Female Group

Table 1 presents the demographic data of the end user group. A total of 116 women participated in the study, 59 in person and 57 online, and their average age was 29.5 ± 5.6 years. Most of the participants had at least a bachelor’s or equivalent degree, except for 42.4% of women in the in-person group. This means that the participants had a high level of education when compared to the average of the Brazilian population, where 20% of adults (25–64 years) have a bachelor’s or equivalent degree [62]. This was expected since the participants were recruited in a university environment. Moreover, approximately half of the users had an income ≥6 times the minimum wage.

Regarding the previous use of products for vaginal administration, 76.4% of women reported that during their lifetime, they had used at least one type of drug product through the vaginal route; semisolid formulations including creams, gels, and ointments were the most frequently used (74.1%). None of the participants reported the usage of vaginal films but 7.8% of the end users stated that they had already used a VR. Palmeira-de-Oliveira et al. (2015) [63] also found that semisolid products were the most common (82%) dosage form used for vaginal delivery among women in Portugal, where 10% of participants had also used a VR. On the other hand, the use of tablets (41.8%) and suppositories (56.5%) was much more frequent in their study compared to the present work (tablets = 14.7%; suppositories = 2.6%).

No statistical difference was observed between the in-person and online samples regarding age, household income, and previous use of vaginal dosage form (*p* > 0.05). The level of education was the only difference among the groups, with the online sample having a higher level of education (*p* < 0.001).

#### 3.2.2. Comfort with Use, Acceptability and Preference

The vast majority of women (75.9%) stated that they would feel comfortable using some of the 3D-printed VR designs. The perception of comfort was higher for the in-person than the online group (84.8% vs. 66.7%; χ^2^ = 13.802; *p* = 0.003; V = 0.35). From a practical point of view, this difference was moderate. This is an interesting finding as it signifies that most women had a positive perception of the 3D-printed VRs and demonstrates their willingness to potentially use them.

According to the survey analysis, most of the women stated that they would use or would definitely use the traditional (56.9%), “Y” (76.8%), and flat circle (76.7%) designs. However, only 31.9% of women confirmed the same for the “M” design. Likewise, the median (1st quartile–3rd quartile) of acceptability for each design was traditional = 4.0 (3.0–4.0), “Y” = 4.0 (4.0–5.0), “M” = 3.0 (2.0–4.0), and flat circle = 4.0 (4.0–5.0). Thus, the traditional, “Y”, and flat circle devices were considered acceptable, whereas “M” was not. As expected, the traditional circle/torus design demonstrated good acceptability. During training, it was clarified to all groups that this design was commercial geometry. Interestingly, the novel “Y” and flat circle designs were also considered acceptable by the women. Although geometry “Y” initially appears to be unusual, when it is folded (Figure 2F), it resembles a tampon, which is an already well-known used product. For the flat circle design, the combination of a geometry similar to the traditional device and a more delicate cross-section could have led to good acceptance. In contrast, the “M” design was the only one that was not well accepted by the end users. Most participants expressed worries that the sharp ends of the device could make insertion difficult or cause injury.

**Figure 3:**
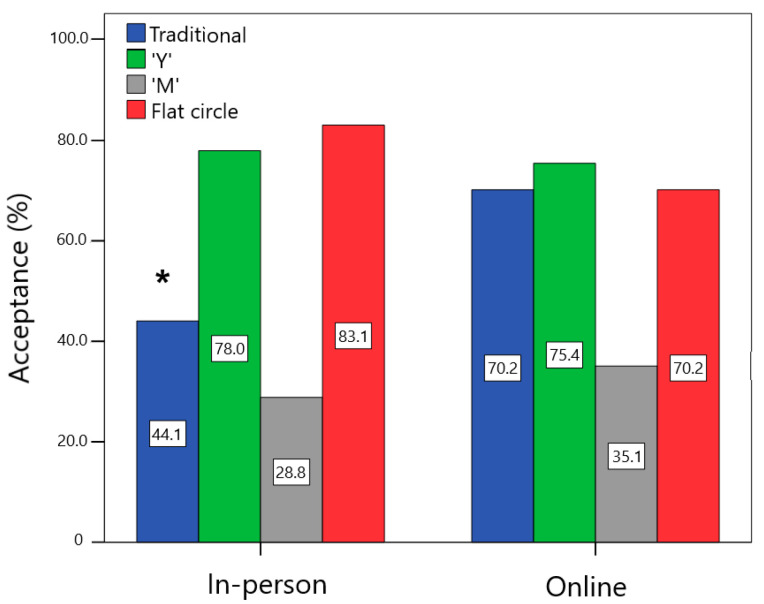
Comparison of the percentage of acceptance (women who would use or would definitely use each device) among the in-person and online users. * Statistically significant difference between groups, *p* < 0.05.

Figure 3 shows that the acceptance for the traditional design was higher for the group that filled out the online form in comparison to the in-person one (χ^2^ = 8.058; *p* = 0.005; V = 0.26). In contrast, there was no difference in acceptance between the groups for “Y” (χ^2^ = 0.104; *p* = 0.75; V = 0.03), “M” (χ^2^ = 0.525; *p* = 0.47; V = 0.07), and flat circle (χ^2^ = 2.691; *p* = 0.10; V = 0.15) designs. For 99.1% of the women, the flexibility of the VR influenced their willingness to use it, with no significant difference between the in-person and online groups (98.3% vs. 100.0%; χ^2^ = 1.595; *p* = 0.44; V = 0.12). All end users (100.0%) stated that they preferred the flexible over the rigid device.

**Figure 4:**
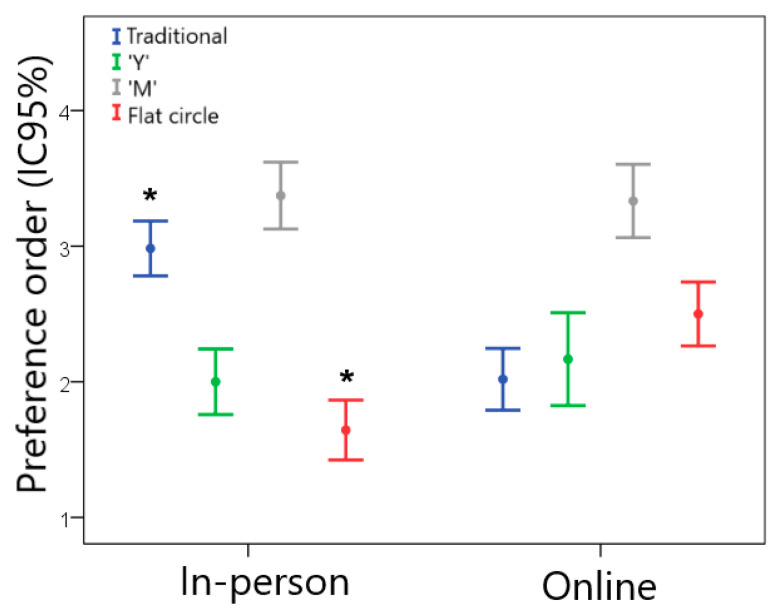
Preference order for women regarding the different VR geometries (1 = most preferred and 4 = least preferred) for the in-person and online groups. * Statistically significant difference between the in-person and online groups, *p* < 0.05.

Figure 4 illustrates the preference order of the end users for the different VR designs in the in-person and online groups. For the in-person sample, the flat circle device was preferred, followed by designs “Y” > traditional > “M”. A slightly different result was found for the online sample, in which the traditional design was the one that women liked the most, followed by designs “Y” > flat circle > “M”. In the comparison between the groups, the traditional design was less preferred by the in-person group than by the online group (Z = −5.480; *p* < 0.001). On the other hand, the in-person group declared a greater preference for the flat circle device compared to the online group (Z = −4.896; *p* < 0.001).

From the results, it is clear that women have different preferences regarding the designs of the 3D-printed VRs and that there is no single preferred geometry. The availability of a greater variety of designs, which is feasible through 3D printing, would be important to meet the preferences of each woman. Moreover, in a scenario where women could choose their preferred geometry from several available options, taking an active role in the decision of their therapeutic treatment could be advantageous since patient participation in healthcare decision making causes improved health outcomes, enhanced quality of life, and the delivery of more appropriate and cost-effective services [64,65].

Three-dimensional printing of medicines has been described as a potential fit for the telemedicine cycle, where the patient undergoes a virtual medical consultation and, based on a remote diagnosis, receives an electronic prescription that guides the design and 3D printing of personalized medicines [66,67]. In this context, it is relevant to evaluate the impact of the method of participation (in person or online) on women’s perception. Overall, the acceptability of the design showed good agreement between the online and in-person results, with only one design (traditional) showing a significant difference between the groups. Regarding the “preference”, two designs (traditional and flat circle) demonstrated a significant difference between the online and in-person groups. This was related to the in-person demonstration of the group where women had the opportunity to observe the details of the geometry, texture, and flexibility of the VRs.

#### 3.2.3. Association between Personal Background and Women’s Acceptability

The acceptability and preference of women regarding vaginal products can be affected by several factors, including those that are related to the product and personal aspects, such as age, socioeconomic and cultural status, and prior experience with the vaginal route of administration [68,69,70]. Therefore, we evaluated whether personal background (age, level of education, household income, previous use of VRs, previous use of at least one vaginal dosage form, and comfort with using 3D-printed VRs) was associated with the acceptance of each VR design.

Table 2 shows the variables associated with the acceptance of each design by the end users. The previous use of VRs and the use of at least one vaginal dosage form by women were associated with greater acceptance of the traditional design. This is consistent with the literature, as the majority of the women who have used VRs before considered it acceptable [71]. Since the women generally had a good experience with the marked VR, they were more prone to choose the traditional 3D-printed design. The acceptance of the innovative devices “Y” and flat circle was greater in younger women and the end users who reported a greater perception of comfort using a 3D-printed VR. Finally, the acceptance of device “M” was associated with women’s comfort. The other variables (level of education, income, and device flexibility) were not associated with acceptance (*p* > 0.05).

### 3.3. Evaluation of Gynecologists’ Perceptions

#### 3.3.1. Technical Opinion

The sample of gynecologists included 25 women and 14 men, with an average age of 44.0 ± 11.2 years. The in-person group consisted of 30 physicians, while the online group consisted of 9 physicians. Table 3 depicts the opinions (in terms of descriptive values) of the gynecologists about each design regarding technical parameters (willingness to prescribe, suitability with the vaginal anatomy, level of difficulty for insertion and manipulation, comfort during use, and possible interference during sexual intercourse).

The traditional design would be prescribed by almost 100% of gynecologists. According to most gynecologists, this device is suitable for the vaginal anatomy, it is comfortable to use, the insertion and manipulation are easy, and it does not interfere with sexual intercourse. This result was similar for the novel design flat circle; however, the gynecologists stated this VR is more suitable for the vaginal anatomy, it will be more comfortable for women, and there will be less interference during sexual intercourse. In contrast, the “Y” and “M” designs were not well evaluated by the gynecologists; 41% would prescribe “Y” and just 20.5% would prescribe “M”. For the design “Y”, the physicians believed that its insertion would be easy with a moderate level of difficulty for manipulation. In addition, for the other parameters, they had divided opinions and demonstrated a high percentage of uncertainty (high percentage of “maybe”). Design “M” was considered to have a moderate level of difficulty for both insertion and manipulation. Furthermore, most doctors claimed that “M” is not suitable for the vaginal anatomy, it will not be comfortable to use, and it may interfere with sexual intercourse.

No statistically significant differences were observed in technical parameters (willingness to prescribe, suitability with the vaginal anatomy, level of difficulty for insertion and manipulation, comfort during use, and possible interference during sexual intercourse) between the in-person and online groups (*p* > 0.05). All gynecologists (100%) stated that the flexibility of the VR influenced their willingness to prescribe, and they preferred the flexible device to the rigid device.

#### 3.3.2. Preferences

**Figure 5:**
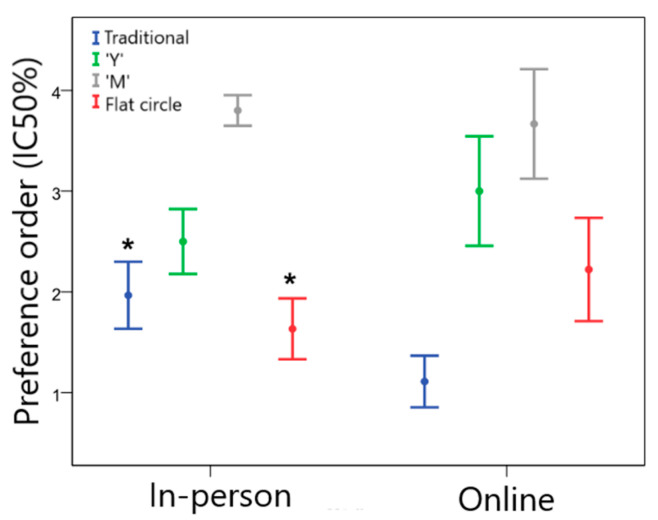
Preference order for gynecologists regarding the different VR geometries (1 = most preferred and 4 = least preferred) for the in-person and online groups. * Statistically significant difference between groups, *p* < 0.05.

The gynecologists’ preference order is presented in Figure 5. According to the data, the flat circle was preferred for the in-person group, followed by designs traditional > “Y” > “M”. For the online group, the preference was for the traditional VR, followed by flat circle > “Y” > “M”.

Among gynecologists, the order of device preference varied between the groups. The traditional design was less preferred by the in-person group than by the online group (Z = −2.832; *p* = 0.007). On the other hand, the in-person group declared a greater preference for the flat circle design compared to the online group (Z = −2.178; *p* = 0.029). Although this difference was observed, there was a clear preference among physicians for traditional and flat circle devices, which is consistent with the results for prescribing intent.

### 3.4. Comparison between the Women’s and Gynecologists’ Perceptions

**Figure 6:**
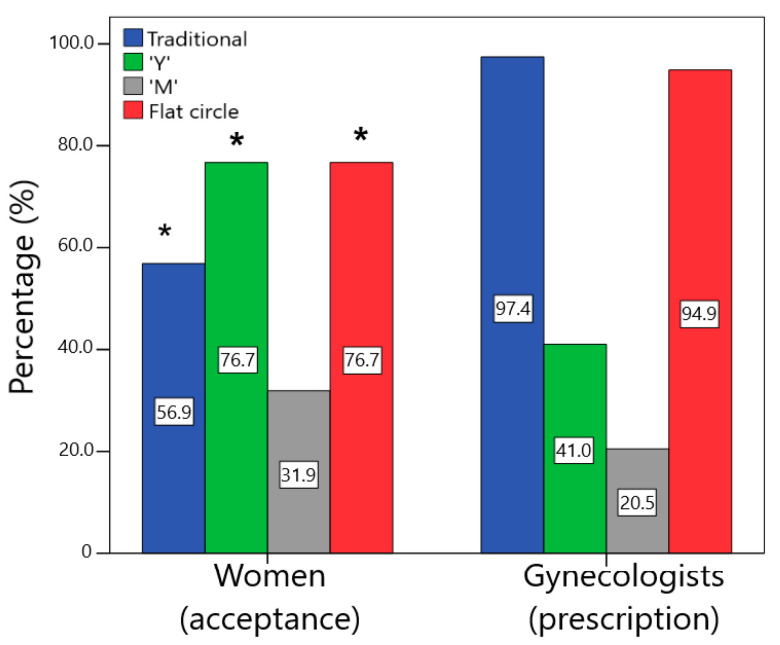
Comparison of the percentage of acceptance (would use or would definitely use) by the women and the prescription (would prescribe or would definitely prescribe) by the gynecologists for the different designs (* statistically significant difference between groups, *p* < 0.05).

Statistical differences were found regarding the acceptance of the geometries between the women and the gynecologists (Figure 6). Almost all physicians would prescribe the traditional design, while only approximately 60% of end users reported that they would use this VR (χ^2^ = 21.727; *p* < 0.001; V = 0.37). Approximately 95% of gynecologists reported that they would prescribe the flat circle, while approximately 80% of women stated that they would use it (χ^2^ = 6.320; *p* = 0.01; V = 0.20). For the design “Y”, approximately 80% of women reported that they would use it, while less than half (41%) of the physicians reported that they would prescribe it (χ^2^ = 17.021; *p* < 0.001; V = 0.33). Finally, the “M” device was the least accepted by users and the one that would be least prescribed by physicians (χ^2^ = 1.836; *p* = 0.17; V = 0.11).

In general, physicians were more conservative than women, with a greater tendency to prescribe the traditional design. However, they also demonstrated a good opinion of the flat circle VR, which is similar to the traditional design. Women, on the other hand, were more open to accepting innovative devices, showing a good perception regarding “Y” and flat circle designs and often preferring these novel shapes over traditional ones. The main difference in the perception of VRs between women and gynecologists was concerning the “Y” device since, despite the differences in the acceptance of traditional and flat circle designs, both were well-evaluated by most of the women and gynecologists. The “Y” design divided the opinion of the gynecologists with a high level of uncertainty, which could indicate that they could prescribe it, but they require more information about the efficacy and safety of this geometry.

## 4. Conclusions

In this study, we conducted a survey analysis on the perception of women and clinicians in regard to the use of 3D-printed personalized vaginal rings with different designs. The survey analysis revealed that 3D-printed VRs were well received by women and gynecologists in the preliminary evaluation. Both traditional and innovative geometries have been accepted by women and gynecologists, although physicians are more conservative. The anticipated acceptability of women regarding the VR designs was affected by their age, perception of comfort, and previous experience with the vaginal route. The women presented multiple preferences towards the geometry, which could indicate that the availability of a greater variety of designs, feasible through 3D printing, could meet the preferences of each woman, improving therapeutic adherence.

## Figures and Tables

**Table 1:** Demographic characteristics of the women participating in the study, stratified by type of participation (in-person sample versus online sample).

Variables	General (*n* = 116)	In Person (*n* = 59)	Online (*n* = 57)	*p* Value	ES
Age (years)	29.5 ± 5.6	28.8 ± 6.1	30.3 ± 4.8	0.13	0.27
Level of education					
Undergraduate education incomplete	30 (25.9%)	25 (42.4%)	5 (8.8%)	<0.001	0.40
Bachelor’s or equivalent	20 (17.2%)	7 (11.9%)	13 (22.8%)		
Specialization	32 (27.6%)	11 (18.6%)	21 (36.8%)		
Master/Doctoral	34 (29.3%)	16 (27.1%)	18 (31.6%)		
Household income					
Up to 3 times minimum wage *	24 (20.7%)	15 (25.4%)	9 (15.8%)	0.37	0.16
3 to 6 times minimum wage	36 (31.0%)	20 (33.9%)	16 (28.1%)		
6 to 12 times minimum wage	36 (31.0%)	15 (25.4%)	21 (36.8%)		
≥12 times minimum wage	20 (17.2%)	9 (15.3%)	11 (19.3%)		
Previous use of vaginal dosage form					
Semisolid ** (yes)	86 (74.1%)	43 (72.9%)	43 (75.4%)	0.75	0.03
Vaginal ring (yes)	9 (7.8%)	4 (6.8%)	5 (8.8%)	0.69	0.04
Suppository (yes)	3 (2.6%)	2 (3.4%)	1 (1.8%)	0.58	0.05
Tablet (yes)	17 (14.7%)	7 (11.9%)	10 (17.5%)	0.39	0.08
Vaginal film	0 (0%)	0 (0%)	0 (0%)	-	-
Never used	27 (23.6%)	15 (25.4%)	12 (21.1%)	0.58	0.05

Values expressed as the mean ± standard deviation and frequency (%); ES: effect size; * minimum wage in Brazil is 1212 BRL/month (229 USD); ** semisolid formulations: cream, gel, and ointment.

**Table 2:** Variables associated with acceptance of each VR geometry by women.

Design Acceptability	Explanatory Variables			
	Previous Use of Vaginal Ring	Prior Use of at Least One Vaginal Dosage Form	Comfort	Age
Traditional				
Would use (*n* = 66)	13.6%	84.8%	4.0 (4.0–5.0)	29.4 ± 4.9
Would not use (*n* = 50)	0.0%	66.6%	4.0 (3.0–5.0)	29.7 ± 6.4
*p* value	0.01 *	0.02 *	0.11	0.75
“Y”				
Would use (*n* = 89)	6.7%	75.3%	4.0 (4.0–5.0)	28.8 ± 5.0
Would not use (*n* = 27)	11.1%	81.5%	4.0 (3.0–4.0)	31.7 ± 6.8
*p* value	0.43	0.50	0.02 **	0.02 #
“M”				
Would use (*n* = 37)	5.4%	75.7%	4.0 (4.0–5.0)	28.6 ± 6.2
Would not use (*n* = 79)	8.9%	77.2%	4.0 (3.0–5.0)	30.0 ± 5.2
*p* value	0.72	0.85	0.007 **	0.21
Flat circle				
Would use (*n* = 89)	6.7%	75.3%	4.0 (4.0–5.0)	28.6 ± 5.0
Would not use (*n* = 27)	11.1%	81.5%	4.0 (3.0–4.0)	32.5 ± 6.3
*p* value	0.43	0.50	0.003 **	0.001 #

Percentages are in relation to the lines; median (1st quartile–3rd quartile); mean ± standard deviation; statistically significant difference, *p* < 0.05; * chi-square test; ** Mann–Whitney test; # Student’s *t*-test.

**Table 3:** Gynecologist opinions (in terms of descriptive values) for technical parameters, stratified by design.

Technical Parameters	Design			
	Traditional	“Y”	“M”	Flat Circle
Willingness to prescribe				
Would prescribe	38 (97.5%)	16 (41.0%)	8 (20.5%)	37 (94.9%)
Would not prescribe	1 (2.5%)	23 (59.0%)	31 (79.5%)	2 (5.1%)
Suitability with the vaginal anatomy				
Yes	31 (79.5%)	18 (46.2%)	7 (17.9%)	36 (92.3%)
No	3 (7.7%)	6 (15.4%)	22 (56.4%)	3 (7.7%)
Maybe	5 (12.8%)	15 (38.5%)	10 (25.6%)	0 (0.0%)
Level of difficulty for insertion *	2.0 (2.0–3.0)	2.0 (2.0–3.0)	3.0 (2.0–4.0)	2.0 (2.0–3.0)
Comfort during use				
Yes	32 (82.1%)	18 (46.2%)	5 (12.8%)	35 (89.7%)
No	2 (5.1%)	4 (10.3%)	20 (51.3%)	0 (0.0%)
Maybe	5 (12.8%)	17 (43.6%)	14 (35.9%)	4 (10.3%)
Level of difficulty for manipulation *	2.0 (2.0–3.0)	3.0 (2.0–3.0)	3.0 (3.0–4.0)	2.0 (2.0–3.0)
Interference during sexual intercourse				
Yes	2 (5.1%)	11 (28.2%)	20 (51.3%)	0 (0.0%)
No	31 (79.5%)	7 (17.9%)	3 (7.7%)	33 (84.6%)
Maybe	6 (15.4%)	21 (53.8%)	16 (41.0%)	6 (15.4%)

Data presented as frequencies (percentages) and median (1st quartile–3rd quartile). * 1 = “very easy”; 5 = “very difficult”.

## Data Availability

Data will be made available on request.

## Supplementary Materials

The following supporting information can be downloaded at: https://www.mdpi.com/article/10.3390/pharmaceutics15092302/s1, Figure S1: Optical images of the different VR geometries produced by FDM technology with PLA filament.
