# Supplementary material for: Three-Dimensionally Printed Vaginal Rings: Perceptions of Women and Gynecologists in a Cross-Sectional Survey
Source: Pharmaceutics. 2023 Sep 11;15(9):2302. doi: 10.3390/pharmaceutics15092302 (PMC10537249; doi:10.3390/pharmaceutics15092302)
Supplement: Supplementary file 1 [file pharmaceutics-15-02302-s001.zip › pharmaceutics-2570371-supplementary.pdf]

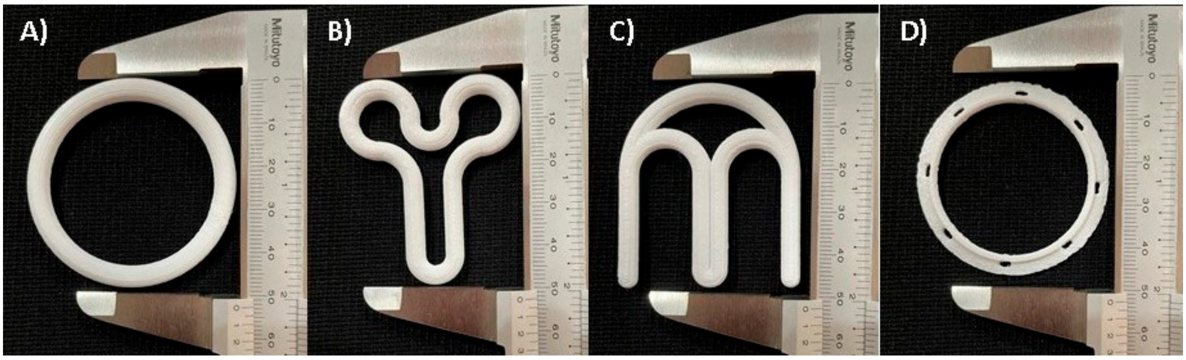

Figure S1. Optical images of the different VR geometries produced by FDM technology with PLA filament.
